# The lifespan and healthspan extending effects of ellagic acid in *Caenorhabditis elegans* require an intact insulin/IGF-1 signaling pathway

**DOI:** 10.3389/fragi.2026.1811330

**Published:** 2026-05-29

**Authors:** Lipeng Xu, Jinze Li, Anran Li, Jixian Chen, Liping Wang, Yaming Shan, Xianbin Cheng

**Affiliations:** 1 National Engineering Laboratory for AIDS Vaccine, School of Life Sciences, Jilin University, Changchun, Jilin, China; 2 Key Laboratory for Molecular Enzymology and Engineering, The Ministry of Education, School of Life Sciences, Jilin University, Changchun, Jilin, China; 3 Department of Thyroid Surgery, The Second Hospital of Jilin University, Changchun, Jilin, China

**Keywords:** *ellagic acid*, C. *elegans*, lifespan, healthspan, insulin/IGF-1 signaling pathway

## Abstract

**Introduction:**

Ellagic acid (EA) is a naturally occurring polyphenolic antioxidant speculated to influence aging, but its precise biological mechanisms remain poorly understood.

**Methods:**

Using Caenorhabditis elegans as a model system, we investigated the anti-aging properties of EA. Lifespan assays were performed at multiple concentrations, and healthspan parameters including locomotor activity, lipofuscin accumulation, and reactive oxygen species (ROS) levels were assessed under thermal and oxidative stress. Genetic requirement was examined using loss-of-function mutants of daf-16, hsf-1, hlh-30, and skn-1. Gene expression was analyzed by qRT-PCR and reporter strains, and global transcriptional changes were mapped by RNA-sequencing.

**Results:**

EA extended lifespan and preserved key healthspan features, including improved locomotor activity, reduced intestinal lipofuscin accumulation, and decreased ROS levels under stress conditions. The lifespan-extending effects of EA required daf-16, hsf-1, hlh-30, and skn-1, as EA failed to extend lifespan in these mutants. EA also enhanced the expression of downstream cytoprotective genes. Complementary RNA-sequencing revealed broader transcriptional remodeling, including metabolic pathways.

**Discussion:**

These findings position EA as a modulator of a coordinated transcriptional network—rather than a single pathway—involving the insulin/IGF-1 signaling pathway and interconnected stress-response networks to promote longevity and stress resistance.

## 1 Introduction

Aging is a universal biological phenomenon experienced by all organisms, and its progression is closely accompanied by a rising burden of chronic disorders, including malignancies, cardiovascular conditions, and metabolic diseases such as diabetes (Zhu et al., 2020). This growing health challenge has intensified efforts to identify interventions capable of slowing aging or mitigating its consequences (Gems, 2014). Beyond behavioral adjustments—such as quitting smoking, moderating alcohol consumption, improving dietary patterns, and maintaining adequate sleep—accumulating evidence suggests that antioxidant supplementation may offer an additional avenue to counteract aging-related decline (Godic, 2019).

Ellagic acid (EA), also known as tricarboxylic formic acid, is a naturally occurring polyphenol abundant in many fruits, nuts, and vegetables. It has been reported to exert diverse biological activities, most notably anti-tumor, anti-inflammatory, and stress-mitigating effects (Zeb, 2018; Bai et al., 2022). Despite these well-recognized properties, its potential role in modulating aging and the molecular basis of such effects remain insufficiently characterized. Since excessive production of reactive oxygen species (ROS) is a major driver of cellular dysfunction and organismal aging (Finkel et al., 2000), antioxidants have been proposed as promising candidates for aging intervention (Sadowska-Bartosz and Bartosz, 2014). Given EA’s intrinsic antioxidant capacity and documented function in stress protection, we postulated that EA might alleviate aging by reducing free radical accumulation.

Despite these promising properties, whether EA directly modulates aging-related pathways and which specific genetic regulators are required remain largely unknown. In this study, we systematically investigated the anti-aging activity of ellagic acid using *Caenorhabditis elegans* and tested the hypothesis that its effects involve conserved longevity pathways, particularly the insulin/IGF-1 signaling (IIS) network and its associated transcription factors.

*Caenorhabditis elegans* (*C. elegans*) serves as a powerful model for aging research due to its short lifespan, ease of cultivation, well-defined genetics, and substantial conservation of gene regulatory pathways with humans—over 65% of its genes share functional homology (Kim and Lee, 2019; Baumeister and Ge, 2002). In the present study, we used *C. elegans* to systematically evaluate the anti-aging activity of EA and to explore its underlying mechanisms.

In *C. elegans*, the insulin/IGF-1 signaling (IIS) pathway is a central regulator of longevity and stress response. The *daf-2* gene encodes the insulin/IGF-1 receptor, whose reduced activity extends lifespan via the downstream transcription factor *daf-16* (FOXO homolog), which activates cytoprotective and metabolic genes. Other transcription factors such as *hsf-1* (heat shock factor 1), *hlh-30* (TFEB homolog, involved in autophagy and lysosomal function), and *skn-1* (NRF2/CNC homolog, governing oxidative stress responses) also interact with the IIS pathway to modulate aging and stress resilience. Given that EA exhibits antioxidant and stress-protective properties, we hypothesized that its lifespan-extending effects might require these conserved longevity-associated genes, and we therefore systematically examined their roles in EA-mediated longevity.

In *C. elegans*, loss-of-function mutations in daf-2 extend lifespan substantially. Testing EA in such a long-lived background is a standard approach to determine whether EA acts through the same pathway as reduced IIS: if EA extends lifespan by inhibiting daf-2, it should not further extend the already-maximized lifespan of daf-2 mutants (epistasis analysis). Conversely, if EA acts independently of IIS, it would still extend lifespan in daf-2 mutants. Therefore, the lack of additional extension in daf-2 (e1370) mutants (see Results) indicates that EA and reduced IIS share a common mechanism.

## 2 Materials and methods

### 2.1 *Caenorhabditis elegans* strains and maintenance

All *C. elegans* strains used in this study were cultured at 20 °C on standard nematode growth medium (NGM) plates seeded with *E. coli* OP50 as a food source, unless otherwise specified for mutant maintenance. The following strains were utilized (Table 1).

**TABLE 1:** *Caenorhabditis elegans* strains.

Strain	Genotype	Description/Source
N2	Wild-type	Bristol reference strain
DA1116	eat-2 (ad1116)II	Dietary restriction model
CF1553	muls84	sod-3
CL2166	dvls19	gst-4
TJ375	gpIs1	hsp-16.2
DAF-2	Mutant daf-2 (e1370) III	Insulin/IGF-1
DAF-16	Mutant daf-16 (mgDf50) I	FOXO transcription factor mutant
HSF-1	Mutant hsf-1 (sy441) I	Heat shock transcription factor mutant
HLH-30	Mutant hlh-30 (tm1978) IV	TFEB ortholog mutant
SKN-1	Mutant skn-1 (zu67) IV	Nrf2/CNC ortholog mutant

### 2.2 Preparation of ellagic acid and *E. coli* OP50

Ellagic acid (EA, 96%, Aladdin, Shanghai, China) was dissolved in dimethyl sulfoxide (DMSO) to prepare a 200 mM stock solution containing 2% DMSO. The solution was sterilized by filtration through a 0.2 µm membrane. For experimental use, the EA stock was mixed with cultures of *E. coli* OP50 to obtain working suspensions containing 0.2% DMSO and final EA concentrations of 50 μM, 100 μM, or 200 µM.

### 2.3 Lifespan experiment

Lifespan assays were performed on standard nematode growth medium (NGM) plates seeded with *E. coli* OP50 as the food source. Age-synchronized populations were obtained by hypochlorite treatment of gravid adults.

For wild-type (N2) and non-temperature-sensitive mutant strains: Synchronized L1 larvae were placed on NGM plates and raised at 20 °C until they reached the young adult (day 0 of adulthood) stage. At this point, they were transferred to fresh NGM plates containing either vehicle control (0.2% DMSO) or EA at the indicated concentrations (50 μM, 100 μM, or 200 µM). All plates were supplemented with 50 µM 5-fluoro-2′-deoxyuridine (FUdR, Aladdin, Shanghai, China) to prevent progeny development.

For the temperature-sensitive mutant daf-2 (e1370): Worms were cultured at the permissive temperature of 16 °C from egg until the L4 larval stage. They were then synchronized to the young adult stage at 16 °C before being transferred to experimental plates (containing EA or control) and shifted to the restrictive temperature of 20 °C for the duration of the lifespan assay. FUdR (50 µM) was included in all plates.

For other mutant strains (daf-16 (mgDf50), hsf-1 (sy441), hlh-30 (tm1978), skn-1 (zu67)): The assay protocol followed that of the wild-type N2 worms, conducted entirely at 20 °C.For all assays, at least 70 animals were included per experimental group. Worms were transferred to fresh treatment plates every second day to maintain consistent exposure and food supply. Animals were scored as dead when they failed to respond to gentle touch with a platinum wire. Worms that crawled off the plate, exhibited internal hatching (bagging), or died from desiccation on the plate walls were censored from the analysis. Survival was monitored daily until all worms had died. Each lifespan experiment was performed in at least three independent biological replicates. Statistical analysis was performed using the log-rank (Mantel-Cox) test in GraphPad Prism 8. All strains used are listed in Supplementary Table S1.

### 2.4 Antibacterial assay

Each well of a 96-well plate was filled with 100 µL of LB medium containing *E. coli* OP50 with or without EA. This timepoint was recorded as 0 h. The plate was incubated at 37 °C, and bacterial growth was measured every 2 h by recording OD600 using an Infinite 200 Pro microplate reader (Tecan, Switzerland).

### 2.5 Lipofuscin assay

After 10 days of EA exposure, worms were anesthetized on 2% agarose pads. Images were captured with a fluorescence microscope (CX23, Olympus, Tokyo, Japan). Approximately 20 worms per group were examined for blue fluorescence (Ex/Em 350/460 nm). Fluorescence intensity was quantified using ImageJ version 15.2 (Schneider et al., 2012).

### 2.6 Body bend assay

The number of body bends was recorded for 30 s per worm under a stereomicroscope, as described previously (Yang et al., 2018). Thirty worms treated with EA for 3 days, 5 days, or 7 days were transferred to NGM plates without food. Ten microliters of M9 buffer was added to each plate. The number of body bends was recorded for each worm.

### 2.7 Pharyngeal pumping assay

Approximately 20 worms were treated with or without 100 µM EA for 3 days, 5 days, or 7 days on NGM plates. Pharyngeal pumping was recorded for 10 s using a COIC stereomicroscope (BK1201, Chongqing, China).

### 2.8 Reproduction assay

Synchronized young adult worms at the L4 larval stage were individually transferred to fresh NGM plates seeded with *E. coli* OP50, containing either 100 µM EA or vehicle control. This time point was designated as Day 0 of the reproductive period. To separate the progeny from the parents, each worm was transferred to a new treatment plate every 24 h for the duration of its egg-laying period (typically 3–4 days). The original plates, now containing the laid eggs, were incubated at 20 °C for an additional 48 h to allow the progeny to develop. Progeny counts were performed when the offspring had developed to the L2 or L3 larval stage, at which point they were clearly visible and could be accurately enumerated under a stereomicroscope. To ensure complete recovery of progeny and mitigate potential loss from eggs being partially embedded in the bacterial lawn, each plate was gently washed with 1 mL of M9 buffer prior to counting, and all suspended larvae were quantified. The total number of progeny produced by each individual worm across all days was summed and recorded as its brood size. Data are presented as the mean brood size per worm for each treatment group.

Approximately 20 individual worms were analyzed per group. The washing step with M9 buffer was performed to ensure retrieval of all progeny that might adhere to the bacterial lawn; this method has been validated in our preliminary experiments showing >95% recovery of larvae. For validation, we compared manual counting with washing-assisted counting in 10 pilot worms, which yielded no significant difference.

### 2.9 Body length and body width assay

Eggs were placed on NGM plates with or without 10 µM EA and marked as the 0 h timepoint. Images were taken at 24, 48, and 96 h. Body length and width were measured using ImageJ version 15.2. Approximately 30 worms were measured per group at each time point. Worms were maintained at 20 °C throughout development, and imaging was performed at room temperature (22 °C–23 °C) within 10 min of mounting.

### 2.10 Thermal shock assay

Groups of 20–30 L4-stage worms were cultured on NGM plates containing 50 µM FUdR with or without 100 µM EA for 5 days (Kumsta et al., 2017). Worms were then exposed to 35 °C for 6 h, followed by a 12-h recovery period. Survival was scored afterward. Worms were scored as dead if they failed to respond to gentle head touch with a platinum wire and exhibited no pharyngeal pumping for 10 s.

### 2.11 Oxidative stress assay

Worms synchronized to the L4 stage were transferred to fresh NGM plates containing either 100 µM EA or a vehicle control and treated for 24 h (Jia et al., 2018). After this pretreatment, worms were placed onto new NGM plates containing 50 mM paraquat (Aladdin, Shanghai, China). Crucially, to assess the protective effect of EA against oxidative stress, worms from the EA pretreatment group were transferred to paraquat plates that also contained 100 µM EA, while control worms were transferred to paraquat plates without EA. This setup directly tested whether continuous EA exposure could enhance survival under oxidative stress. Fifty worms were monitored in each group. Survival was recorded every 24 h. Throughout the assay, worms were examined daily and moved to fresh treatment plates every second day. Animals that failed to respond to gentle head stimulation were scored as dead.

### 2.12 Quantification of reactive oxygen species (ROS)

ROS levels were measured using 2,7-dichlorofluorescein diacetate (DCFH-DA, Meilunbio, China; Ex/Em 470/550 nm) (Ogawa et al., 2016). Worms treated with EA for 5 days were washed off NGM plates using M9 buffer, and additional washes were performed to completely remove OP50 bacteria. The animals were then incubated with 100 µM DCFH-DA for 30 min at 35 °C or with 50 mM paraquat for 6 h. Fluorescence intensity was quantified using ImageJ version 15.2. Background fluorescence was subtracted for all images. The entire body of each worm was selected as the region of interest, and integrated density values were used to calculate relative fluorescence intensity. Approximately 30 worms were analyzed per group for ROS quantification.

Imaging was performed using a fluorescence microscope (Olympus X71) with fixed exposure time (200 ms) and gain (1×). Quantification was performed blinded to treatment. Background fluorescence was subtracted using a region outside the worm. Fluorescence intensity was normalized to worm area. Dye loading variability was controlled by parallel staining of all groups and using the same batch of DCFH-DA.

### 2.13 Fluorescence imaging

Transgenic strains CF1553, CL2166 and TJ375 were treated with or without 100 µM EA for 5 days. Worms were anesthetized with 10 mM levamisole (Aladdin, Shanghai, China) and mounted on 2% agarose pads. Green fluorescent protein expression was visualized using a fluorescence microscope (Olympus X71, Tokyo, Japan). Each experimental group contained 20 to 30 worms. Imaging was performed with excitation at 470 nm and emission captured at 550 nm. Fluorescence intensity was quantified using ImageJ version 15.2 after subtraction of background signals. The entire body of each worm was evaluated, and integrated density measurements were used to determine the relative fluorescence level.

### 2.14 RNA sequencing

N2 worms treated with 100 µM EA or vehicle control for 5 days were collected in three biological replicates per condition. Total RNA was extracted using TRIzol. Libraries were prepared using NEBNext UltraTM RNA Library Prep Kit for Illumina and sequenced on NovaSeq 6000 (PE150). Reads were trimmed with Trimmomatic (v0.39) and aligned to the *C. elegans* genome (WBcel235) using STAR (v2.7). Gene-level counts were obtained with featureCounts (v2.0). Differential expression analysis was performed using edgeR (v3.34) on raw counts, normalized by TMM. Genes with FDR < 0.01 and |log2FC| > 1 were considered differentially expressed. Raw data have been deposited in GEO under accession GSEXXXXX (to be released upon publication). PCA and sample correlation plots are provided in Supplementary Figure S2.

### 2.15 Quantitative real-time polymerase chain reaction (RT-qPCR) assay

Total RNA was isolated from approximately 2,000 worms treated with EA for 5 days using TRIzol reagent (TransGen Biotech, Beijing, China). Complementary DNA was synthesized with a qRT-PCR kit (Bimake, Houston, TX, USA). RT-qPCR reactions were carried out using the Prism 7500 Real-Time PCR System (ABI, USA) with SYBR PCR reagents (Bimake, Houston, TX, USA). Each reaction contained 0.5 µM primers and 1 µL cDNA in a final volume of 20 µL. Relative gene expression levels were calculated using the 2^−ΔΔCT^ method. The gene act-1 was used as the internal reference. Primer sequences are listed in Supplementary Table S2.

### 2.16 Statistical analysis

All experiments were performed with at least three biological replicates, each consisting of independent worm cohorts on different days. For lifespan assays, at least 70 worms per group per replicate were used. Survival curves were analyzed using the log-rank (Mantel-Cox) test, with hazard ratios (HR) and 95% confidence intervals (CI) reported where applicable. For quantitative assays (e.g., body bends, fluorescence), data are presented as mean ± SD. Comparisons between two groups were performed using two-tailed Student’s t-test after verifying normality (Shapiro-Wilk test) and homogeneity of variance (F-test). For multiple comparisons (e.g., different time points or doses), we applied the Holm–Šídák correction for family-wise error rate. For all tests, *p < 0.05 was considered significant. Blinding was applied during lifespan scoring and image quantification.

## 3 Result

### 3.1 Effect of EA on the lifespan of *Caenorhabditis elegans*

**FIGURE 1:**
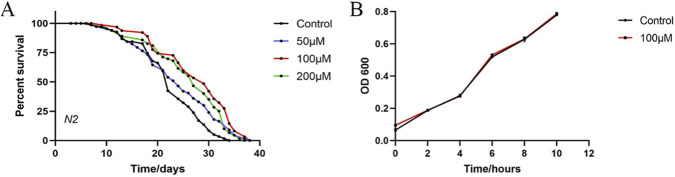
Effects of EA on lifespan in *Caenorhabditis elegans* and bacterial growth in *E. coli* OP50. **(A)** EA extended *Caenorhabditis elegans* lifespan. Wild-type L4 larvae were treated with 0, 50, 100, 200 μM NGM at 20 °C. 100 μM EA treatment showed the most significant lifespan extension effect in *Caenorhabditis elegans*, n = 3 (70 individuals per group), Kaplan–Meier survival analysis with Log-Rank test. **(B)** EA did not affect the growth rate of *E. coli* OP50, which was detected by OD600. Data were analyzed by Student’s t-test using GraphPad Prism 8. Values were presented as mean ± SD.

To determine whether ellagic acid (EA) exerts anti-aging activity in *C. elegans*, worms were exposed to 50 μM, 100 µM or 200 µM EA at 20 °C. Among these treatment groups, 100 µM EA produced the strongest extension of mean lifespan, with an increase of approximately 24.06% compared with the control group (****p < 0.0001) (Figure 1A; Supplementary Table S3). Because bacterial proliferation can influence worm survival, we further examined whether EA affected the growth of *E. coli* OP50, the standard food source for *C. elegans*. OP50 was cultured in LB medium with or without 100 µM EA, and bacterial growth was monitored. The resulting growth curves indicated that EA did not significantly alter OP50 proliferation (*p* > 0.05) (Figure 1B). Based on these observations, the 100 µM concentration of EA, which produced the most pronounced lifespan extension, was used for the remaining experiments.

### 3.2 EA improved the healthspan of *Caenorhabditis elegans*

**FIGURE 2:**
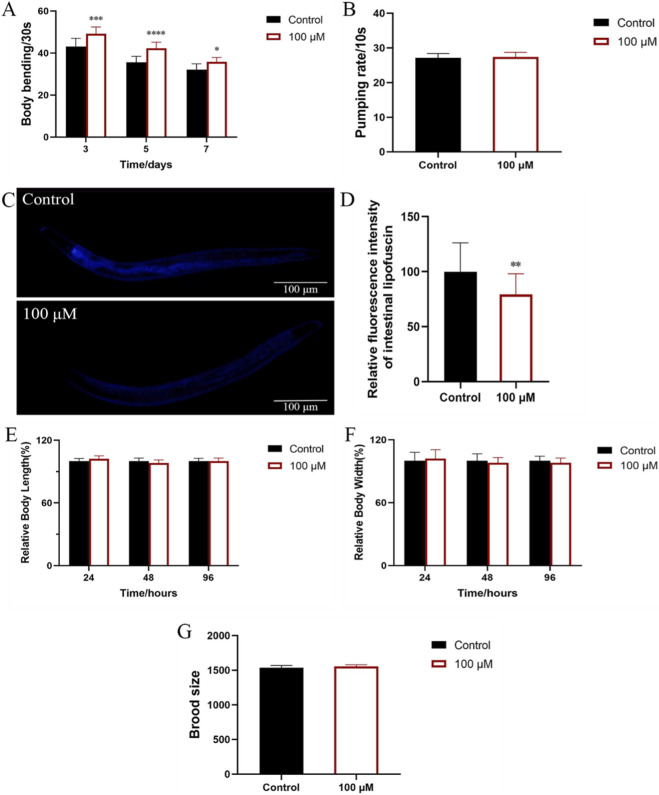
EA partly promoted healthspan of *Caenorhabditis elegans*. **(A)** Effect of EA on body bending rate in N2 worms. **(B)** Effect of EA on pharyngeal pumping rate in N2 worms. **(C,D)** Effect of EA on intestinal lipofuscin in N2 worms. **(E,F)** Effect of EA on body size in N2 worms. **(G)** EA did not affect reproduction in N2 worms. Data were analyzed by Student’s t-test using GraphPad Prism 8. Values were presented as mean ± SD. ****p < 0.0001, ***p < 0.001, **p < 0.01.

We operationally define “healthspan” as the preservation of locomotor function, reduced intestinal lipofuscin accumulation, and enhanced stress resistance, without necessarily affecting all age-related parameters. Day 5 was selected for most assays because it showed the maximal improvement in body bends (Figure 2A) and precedes the onset of major age-related decline in N2 worms under our culture conditions. Physiological decline is a characteristic feature of aging in *C. elegans* (Kumar et al., 2010, Honnen et al., 2012). Several parameters, including locomotion, pharyngeal pumping, intestinal lipofuscin accumulation, reproductive output and overall morphology, are known to deteriorate with age (Han et al., 2017; Kimura et al., 1997; Sun et al., 2016). To evaluate whether EA could improve these age-associated phenotypes, we examined multiple healthspan indicators.

Locomotion was quantified first. Worms treated with 100 µM EA for 3, 5 or 7 days were transferred to M9 buffer, and the number of body bends within 30 s was recorded. EA treatment significantly enhanced locomotor activity, with the strongest effect observed on day 5 (****p < 0.0001) (Figure 2A). Day 5 was therefore selected as the optimal time point for subsequent physiological assays.

Pharyngeal pumping, another well-established marker of nematode aging, was next assessed. EA treatment did not significantly change the pumping frequency (Figure 2B).

Lipofuscin, an autofluorescent pigment that progressively accumulates in the intestine and is widely used as an aging biomarker (Sun et al., 2016; Koubova and Guarente, 2003; Hosokawa et al., 1994; Gerstbrein et al., 2005), was then measured. EA treatment markedly reduced intestinal lipofuscin fluorescence in adult worms (Figures 2C,D), indicating an improvement in age-related oxidative damage.

To determine whether EA influenced growth or reproduction, we analyzed body length, body width and brood size in worms treated with 100 µM EA. No significant differences were observed in body size (Figures 2E,F) or reproductive output (Figure 2G), suggesting that EA did not interfere with development or fertility.

Taken together, these findings indicate that EA mitigated several aging-associated physiological declines and contributed to the maintenance of healthy lifespan in *C. elegans*.

### 3.3 EA can enhance stress resistance and inhibited ROS accumulation of *Caenorhabditis elegans*

**FIGURE 3:**
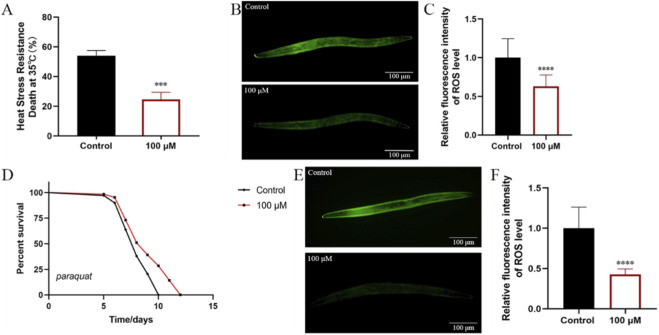
EA improved heat and oxidative stress resistance of *Caenorhabditis elegans*. **(A)** The average death rate of N2 worms cultured with 100 µM EA at 35 °C. **(B,C)** Effects of EA on ROS levels in N2 worms which treated after 35 °C heat stress. **(D)** The lifespan of N2 worms cultured with 100 µM EA and 50 mM paraquat. **(E,F)** Effects of EA on ROS levels in N2 worms which treated after 50 mM paraquat 6 h. Statistical analysis was performed by Student’s t-test or the log-rank test by GraphPad Prism 8. Values were presented as mean ± SD, ***p < 0.001, ****p < 0.0001.

Lifespan extension in *C. elegans* is frequently linked to improved resistance to environmental stressors (Jiang et al., 2021). To examine whether ellagic acid (EA) enhances stress tolerance, L4-stage worms were exposed to 100 µM EA for 5 days and subsequently challenged with paraquat or heat stress at 35 °C. Worms treated with EA displayed markedly improved survival under both stress conditions (Figures 3A,D).

**FIGURE 4:**
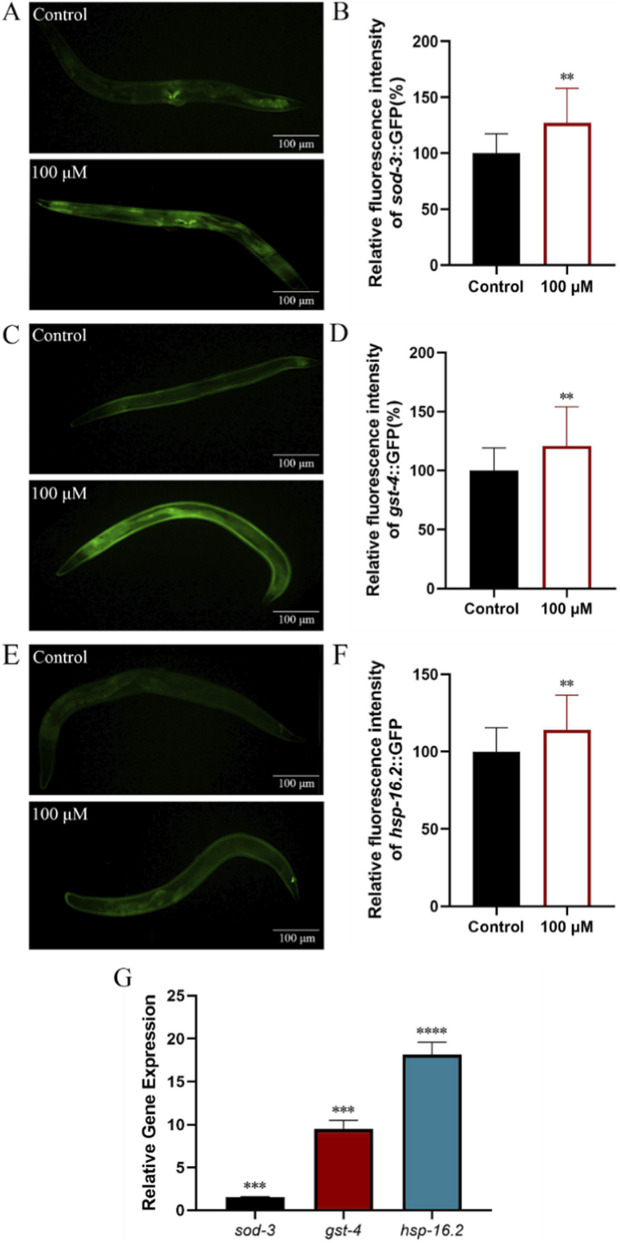
Effect of EA on anti-stress genes expression level. **(A,B)** EA significantly induced the expression of SOD-3::GFP. **(C,D)** EA significantly induced the expression of GST-4::GFP. **(E,F)** EA significantly induced the expression of HSP-16. 2::GFP after incubation at 37 °C for 30 min. **(G)** EA significantly activated the mRNA expression level of sod-3, gst-4 and hsp-16.2. The images were analyzed with ImageJ software and numerical data were analyzed by Student’s t-test using GraphPad Prism 8. Values were presented as mean ± SD, **p < 0.01, ***p < 0.001, ****p < 0.0001.

**FIGURE 5:**
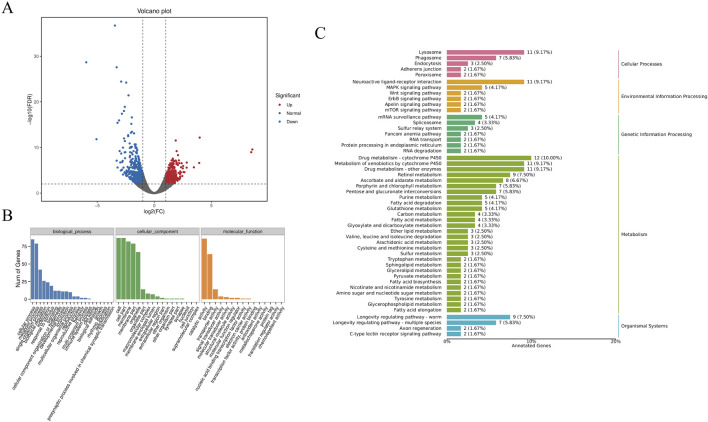
The genome-wide transcriptional change of N2 worms which after treatment with 100 μM EA. **(A)** Volcano map showed the regulated genes that, there were 298 genes upregulated and 517 genes downregulated in EA-treated, compared to control group. **(B)** KEGG pathways enriched by the differentially expressed genes in N2 worm. **(C)** The GO analysis illuminates the biological process, molecular function and cellular component of the differentially expressed genes in N2 worms.

Paraquat exposure and elevated temperature are known to increase reactive oxygen species (ROS) in worms, leading to oxidative damage. To determine whether EA influences ROS accumulation, DCFH-DA staining was used to quantify ROS levels. EA treatment significantly lowered ROS fluorescence under both heat-induced and paraquat-induced stress (Figures 3B,C,E,F). This reduction in ROS levels is likely mediated, at least in part, by EA’s ability to activate endogenous stress-response pathways, including daf-16, skn-1, and hsf-1, as shown by the increased expression of their target genes (see Figures 4, 5). While EA possesses intrinsic radical-scavenging activity, the genetic requirement for these transcription factors indicates that its effects are not solely due to direct chemical antioxidant action. In summary, EA strengthened the ability of worms to cope with stress and reduced the buildup of free radicals, suggesting that activation of stress-related pathways may contribute to its lifespan-extending effects.

### 3.4 EA extends lifespan through the insulin/IGF-1 signaling pathway and requires daf-16

**FIGURE 6:**
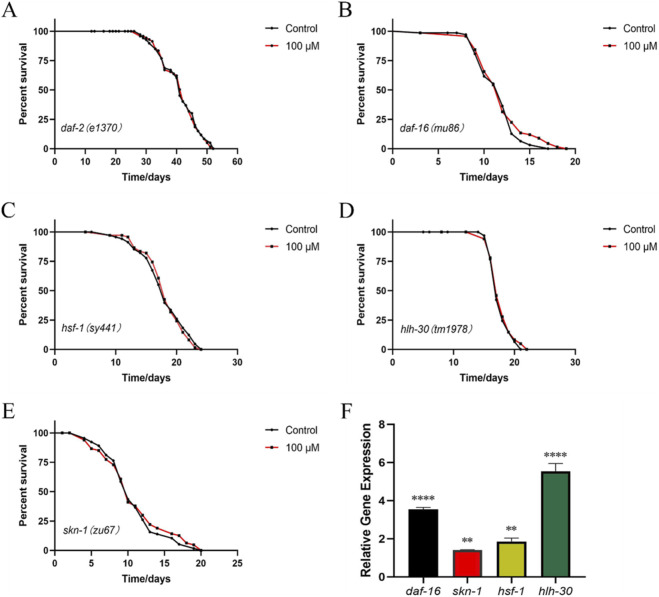
Mechanism of EA-mediated longevity. **(A)** Effect of EA-treated on lifespan in daf-2 (e1370) worms. **(B)** Effect of EA-treated on lifespan in daf-16 (mgDf50) worms. **(C)** Effect of EA-treated on lifespan of hsf-1 (sy441) worms. **(D)** Effect of EA treatment on lifespan of hlh-30 (tm1978) worms. **(E)** Effect of EA-treated on lifespan in skn-1 (zu67) worms. **(F)** daf-16,skn-1,hsf-1,hlh-30 levels in worms treated with 100 µM EA. Statistical analysis of the lifespan was performed using GraphPad Prism8, and p values were calculated by the log-rank test. Numerical data were analyzed by Student’s t-test and values were presented as mean ± SD, **p < 0.01, ****p < 0.0001.

The insulin/IGF-1 signaling (IIS) pathway is a conserved regulator of lifespan and stress resistance across species (Zafrilla et al., 2001). In *C. elegans*, the daf-2 gene encodes the IGF-1 receptor, and its reduced activity extends lifespan via the downstream transcription factor DAF-16/FOXO (Lapierre and Hansen, 2012; Murphy et al., 2003; McElwee et al., 2003). To determine whether EA requires core IIS components for its pro-longevity effect, we performed lifespan assays using daf-2 (e1370) and daf-16 (mgDf50) mutants. EA treatment did not extend the lifespan of either mutant strain (Figures 6A,B; Supplementary Table S3). These results indicate that intact IIS signaling, including both daf-2 and daf-16, is necessary for EA-mediated lifespan extension. However, as shown below, EA also requires other transcription factors (hsf-1, hlh-30, skn-1) that operate in parallel stress-response pathways, suggesting that EA engages a broader regulatory network rather than acting exclusively through IIS.

We next asked whether EA influences daf-16 expression. qRT-PCR showed that EA significantly increased daf-16 mRNA levels (Figure 6F). While this transcriptional upregulation is consistent with DAF-16 involvement, increased transcript levels do not directly demonstrate functional activation of the transcription factor (e.g., nuclear translocation or target gene binding). Nevertheless, the loss-of-function data firmly establish that daf-16 is required for EA’s longevity effect.

### 3.5 EA requires hsf-1 to extended the lifespan of *Caenorhabditis elegans*

The transcription factor hsf-1 regulates heat shock responses, larval development, and stress resistance in *C. elegans*. Higher hsf-1 expression is associated with increased stress tolerance and extended lifespan (Zarse et al., 2012). To test whether hsf-1 is necessary for EA’s effect, we used the hsf-1 (sy441) mutant. EA treatment did not extend the lifespan of these mutants (Figure 6C; Supplementary Table S3), demonstrating that hsf-1 is required for EA-mediated longevity.

We then examined whether EA alters hsf-1 transcript levels. qRT-PCR revealed a significant increase in hsf-1 mRNA following EA treatment (Figure 6F). However, as with daf-16, increased mRNA levels do not directly prove hsf-1 functional activation, which may also involve post-translational modifications or nuclear localization. Nonetheless, the necessity of hsf-1 is clearly supported by the mutant lifespan data.

### 3.6 EA requires hlh-30 to extended the lifespan of *Caenorhabditis elegans*

hlh-30, the worm homolog of mammalian TFEB, is a central regulator of lysosomal function and autophagy. Previous studies have shown that daf-16 and hlh-30 can act cooperatively to regulate longevity and oxidative stress resistance (Ogg et al., 1997; Settembre et al., 2011). To determine whether hlh-30 is necessary for EA’s effect, we examined the hlh-30 (tm1978) mutant. EA failed to extend the lifespan of these mutants (Figure 6D; Supplementary Table S3), indicating that hlh-30 is required for EA-mediated lifespan extension.

qRT-PCR analysis showed that EA treatment significantly elevated hlh-30 mRNA levels (Figure 6F). While this suggests transcriptional regulation, it does not directly demonstrate HLH-30 functional activation, as TFEB activity is also controlled by phosphorylation and subcellular localization. Nevertheless, the mutant data establish that intact hlh-30 is essential for EA’s pro-longevity effect.

### 3.7 EA requires skn-1 to extended the lifespan of *Caenorhabditis elegans*

SKN-1 is the worm homolog of the mammalian NRF/CNC transcription factor family (An et al., 2003). It regulates detoxification genes and contributes to oxidative stress protection (Oh et al., 2005). To determine whether EA requires skn-1 to prolong lifespan, we used *skn-1* (zu67) mutants, which carry a partial loss-of-function allele. Although EA increased skn-1 mRNA levels (Figure 6F), it did not extend the lifespan of *skn-1* (zu67) mutants (Figure 6E; Supplementary Table S3). These results indicate that EA-mediated lifespan extension depends on skn-1. The residual activity in zu67 alleles may still support some baseline function, but the absence of EA benefit suggests that full SKN-1 activity is required for EA’s longevity effect.

### 3.8 EA enhances the expression of anti-stress genes in *Caenorhabditis elegans*

Within the IIS pathway, downstream targets of DAF-16/FOXO and *SKN-1*/*NRF-2* include genes encoding antioxidant and detoxification enzymes such as *sod-3* and *gst-4* (Navarro-Hortal et al., 2022). To investigate whether EA upregulates these genes, transgenic worms carrying *sod-3::GFP* and *gst-4::GFP* reporters were examined. EA treatment for 5 days significantly increased *sod-3::GFP* fluorescence (Figures 4A,B) and also enhanced *gst-4::GFP* expression (Figures 4C,D). These results indicate that EA promotes the activation of stress-defense genes.

The small heat shock protein *hsp-16.2* plays a central role in the heat stress response in *C. elegans*, and its overexpression has been shown to extend lifespan (Lin et al., 2018). Using a transgenic strain expressing *hsp-16.2::GFP*, we observed a substantial increase in fluorescence following EA treatment (Figures 4E,F). qRT-PCR confirmed that EA upregulated the transcripts of *sod-3*, *gst-4* and *hsp-16.2* (Figure 4G). Collectively, these results demonstrate that EA enhances the expression of multiple genes involved in stress resilience.

### 3.9 Genome-wide transcriptional profiling of N2 worms

To further explore the mechanisms by which EA promotes longevity, transcriptome sequencing was performed in N2 worms treated with EA or vehicle control for 5 days. A total of 815 genes were identified as differentially expressed (FDR < 0.01, Fold Change > 2), including 298 upregulated and 517 downregulated genes (Figure 5A).

KEGG pathway analysis revealed enrichment of several metabolic pathways, including retinol metabolism, purine metabolism, fatty acid metabolism, glutathione metabolism and carbon metabolism (Figures 5B,C). Notably, among the upregulated genes, we identified known targets of DAF-16 (e.g., sod-3, ctl-1, mtl-1) and SKN-1 (e.g., gst-4, gst-7), consistent with our reporter gene assays (Figure 4). Conversely, genes involved in insulin-like peptide production (ins-family) were downregulated, suggesting feedback regulation. These transcriptomic data indicate that EA reconfigures a transcriptional network encompassing IIS, oxidative stress response, and metabolic pathways, rather than a single linear cascade.

## 4 Discussion

Ellagic acid (EA) is a naturally occurring phenolic compound abundant in fruits and nuts, widely recognized for its strong antioxidant activity. Previous studies have shown that EA protects against oxidative injury, inflammation, and neurodegenerative processes, although its role in aging regulation has remained incompletely characterized. In this study, we demonstrated that EA significantly extended the lifespan of *C. elegans* in a concentration-dependent manner, with maximal benefit at 100 µM (Figure 1A; Supplementary Table S3). Importantly, this lifespan extension was not attributable to altered food availability, as EA did not affect the proliferation of OP50 bacteria (Figure 1B). Beyond lifespan extension, EA notably enhanced healthspan-related measures that are known to decline with age. Locomotor activity, an indicator of neuromuscular function, was significantly improved (Figure 2A), and the age-associated accumulation of intestinal lipofuscin, a biomarker of oxidative damage, was reduced (Figures 2C,D). In contrast, EA did not alter body size or brood size (Figures 2E–G), indicating that its effects are not mediated through changes in development or fertility. Interestingly, while pharyngeal pumping rate—another marker of physiological aging—was unaffected by EA (Figure 2B), this does not diminish the importance of the observed improvements in locomotion and lipofuscin levels as indicators of preserved healthspan. Collectively, these results suggest that EA ameliorates specific age-related declines without perturbing essential developmental or reproductive functions.

A major driver of cellular aging is the excessive accumulation of reactive oxygen species (ROS), which damages proteins, lipids and DNA and contributes to various age-related disorders (Davalli et al., 2016). We propose that EA’s well-documented antioxidant and redox-modulating properties serve as the initiating mechanism for its observed biological effects. Consistent with this, EA significantly improved survival under heat and paraquat-induced oxidative stress (Figures 3A,D) and concomitantly reduced ROS levels (Figures 3B,C,E,F). This attenuation of oxidative burden likely creates a cellular environment that favors the activation of conserved cytoprotective signaling networks.

The insulin and insulin-like growth factor signaling (IIS) pathway is a key determinant of longevity and stress resilience. Notably, EA did not extend the lifespan of *daf-2* (*e1370*) or *daf-16* (*mgDf50*) mutants (Figures 6A,B), indicating that the IIS pathway and its central transcription factor *daf-16* are essential for EA-mediated lifespan extension. Enhanced expression of *hsf-1* is also linked to longevity and improved stress tolerance in worms (Zarse et al., 2012). EA increased *hsf-1* transcript levels, yet failed to extend lifespan in the *hsf-1* (*sy441*) mutant (Figure 6C), suggesting that *hsf-1* activity is required for EA function. Evidence from earlier studies shows that *daf-16* and HLH-30/TFEB can act together to support stress resistance and longevity (Settembre et al., 2013). Consistent with this, EA did not extend lifespan in *hlh-30* (*tm1978*) mutants, further indicating that HLH-30 contributes to the EA-induced longevity phenotype.

SKN-1, the worm ortholog of the mammalian NRF/CNC family, regulates phase II detoxification genes and plays an important role in lifespan determination (Oh et al., 2005). When IIS activity is reduced, SKN-1 cooperates with daf-16 to initiate stress-protective transcriptional programs (Tullet et al., 2008; Ewald et al., 2015). Consistent with a role in EA’s action, we found that EA increased *skn-1* expression (Figure 6F). Crucially, EA failed to extend lifespan in *skn-1* (zu67) mutants (Figure 6E), demonstrating that SKN-1 is required for EA-mediated longevity. These findings indicate that SKN-1 is an essential component of EA’s mechanism, likely acting in concert with other factors such as DAF-16, rather than functioning alone.

To further characterize EA-induced molecular responses, we examined the expression of *sod-3*, *gst-4* and *hsp-16.2*, three key stress-response genes downstream of IIS. EA significantly increased SOD-3::GFP, GST-4::GFP and HSP-16.2::GFP levels, and qRT-PCR confirmed upregulation at the mRNA level (Figure 4). These results demonstrate that EA activates a broad anti-stress gene network.

**FIGURE 7:**
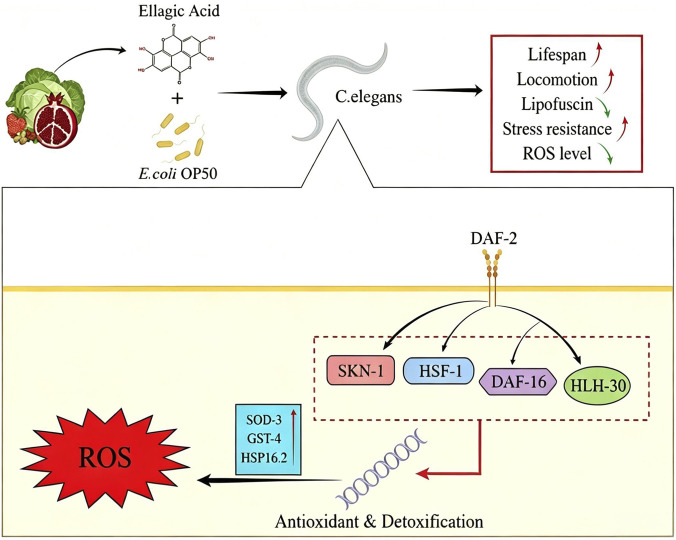
The molecular mechanism by which ellagic acid (EA) delays aging and enhances stress resistance in *Caenorhabditis elegans* (illustrated in Figdraw).

To explore global transcriptional changes, we performed RNA-seq and found that EA treatment altered the expression of 815 genes. A substantial number of these genes were enriched in metabolic pathways, including amino acid metabolism, fatty acid metabolism and glutathione metabolism (Figure 5). The composition of amino acids and related metabolic pathways has strong links to metabolic health and lifespan (Fonte et al., 2008). Branched-chain amino acids and methionine, for example, have been shown to influence longevity in several models (Fonte et al., 2008; Baggs et al., 2023). The enrichment of EA-regulated genes in these pathways suggests that EA may modulate metabolic processes to delay aging. Previous studies propose that lifespan regulation results from interactions among transcripts, proteins and metabolites (Southworth et al., 2009), which aligns with the transcriptomic patterns observed here. The pathway enrichment results therefore imply that metabolic remodeling contributes to the anti-aging effect of EA (Figure 7).

Finally, genes within the MAPK pathway were significantly enriched in EA-treated worms. Because MAPK signaling influences immune responses, we further tested worm resistance to *Pseudomonas aeruginosa* PA14 (Liu et al., 2022). EA substantially improved survival under PA14 challenge and increased expression of the immune-related gene pmk-1 (Supplementary Figure S1; Supplementary Table S4), suggesting that enhanced immune responsiveness may also participate in the longevity benefits conferred by EA. Taken together, our findings demonstrate that EA extends lifespan through coordinated regulation of stress-response pathways, IIS components, immune signaling and metabolic reprogramming.

While our data show that 100 µM EA did not significantly alter the growth curve of *E. coli* OP50 (Figure 1B), this observation does not exclude the possibility that bacteria metabolize EA into bioactive derivatives, or that EA changes the nutritional quality of the bacterial lawn, which could indirectly influence worm physiology. Moreover, the potential contribution of hormesis—a biphasic response where low-dose stress confers benefits—cannot be formally ruled out. Therefore, we interpret our findings as consistent with a direct host-mediated effect of EA, rather than as a definitive demonstration of such action. Future studies using heat-killed or UV-killed bacteria, or pre-treatment of bacteria with EA followed by washing, would help dissect direct versus microbe-mediated mechanisms.

The absence of EA effect in daf-2 (e1370) mutants may partially reflect a ceiling effect, as these animals already exhibit maximally extended lifespan through reduced IIS. However, the requirement of daf-16 in a null background strongly supports that daf-16 is indispensable for EA’s action.

The RNA-seq data presented here provide a global view of EA-induced transcriptional changes. While pathway enrichment analysis highlighted metabolic processes (e.g., glutathione, fatty acid, and amino acid metabolism), a more focused examination revealed that many differentially expressed genes are known downstream targets of daf-16, skn-1, and hsf-1. For example, sod-3 (oxidative stress), gst-4 (detoxification), and hsp-16.2 (proteostasis) were all upregulated, consistent with our reporter assays. Conversely, the downregulation of several ins family insulin-like peptides suggests a possible feedback loop that may amplify the IIS reduction. These observations support a model in which EA orchestrates a multi-branched transcriptional program rather than acting through any single pathway. Future studies using tissue-specific knockouts or chromatin immunoprecipitation will be required to determine the direct versus indirect transcriptional targets of EA.

## Data Availability

The datasets presented in this study can be found in online repositories. The names of the repository/repositories and accession number(s) can be found in the article/Supplementary Material.
