# Supplementary material for: The lifespan and healthspan extending effects of ellagic acid in Caenorhabditis elegans require an intact insulin/IGF-1 signaling pathway
Source: Front Aging. 2026 May 29;7:1811330. doi: 10.3389/fragi.2026.1811330 (PMC13260644; doi:10.3389/fragi.2026.1811330)
Supplement: Supplementary file 1 [file DataSheet1.pdf]

Table S1. All strains used in this study.

Strains	Genotype
N2	*C. elegans* wild isolate.
CB1370	*daf-2(e1370)* III.
GR1307	*daf-16(mgDf50)* I.
EU1	*skn-1(zu67)* IV/nT1 [*unc-?(n754) let-?*] (IV;V).
PS3551	*hsf-1(sy441)* I.
JIN1375	*hlh-30(tm1978)* IV.
TJ375	*gpIs1* [h*sp-16.2p*::GFP].
CF1553	*muIs84* [(pAD76) *sod-3p*::GFP + rol-6(su1006)].
CL2166	*dvIs19* [(pAF15) *gst-4p*::GFP::NLS] III.

Table S2. List of primers used for the quantitative real-time reverse transcription-polymerase chain reaction

Gene name	Primer sequence (5'to3')
act-1(F)	CTACGAACTTCCTGACGGACAAG
act-1(R)	CCGGCGGACTCCATACC
daf-16(F)	CCAGACGGAAGGCTTAAAACT
daf-16(R)	ATTCGCATGAAACGAGAATG
daf-2(F)	CGGTGCGAAGAGAGGATATT
daf-2(R)	TACAGAGGTCGCCGTTACTG
hsf-1(F)	TTGACGACGACAAGCTTCCAGT
hsf-1(R)	AAAGCTTGCACCAGAATCATCCC
hlh-30(F)	CTCATCGGCCGGCGCTCATC
hlh-30(R)	AGAACGCGATGCGTGGTGGG
skn-1(F)	AGTGTCGGCGTTCCAGATTTC
skn-1(R)	GTCGACGAATCTTGCGAATCA
sod-3(F)	AGCATCATGCCACCTACGTGA

sod-3(R)	CACCACCATTGAATTTCAGCG
gst-4(F)	TCCGTCAATTCACTTCTTCCG
gst-4(R)	AAGAAATCATCACGGGCTGG
hsp-16.2(F)	CTGCAGAATCTCTCCATCTGAGTC
hsp-16.2(R)	AGATTCGAAGCAACTGCACC
pmk-1(F)	CCGACTCCACGAGAAGGATA
pmk-1(R)	TCAGCAGCACAAACAGTTCC

Table S3.Effects of EA on the lifespan of N2 and mutant C.elegans. *p* value compared to the control (0 μM) group.

Genotype	Treatment	Mean (Days)	±SD	Percent Change （%）	*p* value (log-rank significance)
N2	0 μM	22.05	3.73		
	50 μM	23.73	7.94	7.63	0.0261
	100 μM	27.35	7.21	24.06	<0.0001
	200 μM	25.97	7.17	17.8	<0.001
daf-2	0 μM	40.70	6.53		
	100 μM	40.71	6.40	0.02	0.7926
daf-16	0 μM	11.45	11.82		
	100 μM	2.28	2.74	3.3	0.3305
hsf-1	0 μM	17.76	3.73		
	100 μM	17.89	3.30	0.7	0.8237
hlh-30	0 μM	17.59	1.52		
	100 μM	17.67	1.71	0.4	0.5141
skn-1	0 μM	10.52	3.73		
	100 μM	10.64	4.28	1.2	0.5627

Figure S1. Effects of EA on C.elegans immune ability. (A)The survival percentage of N2 worms cultured with *Pseudomonas aeruginosa* under treatment with 100 μM of EA;(B) EA significantly activated the mRNA expression level of *pmk-1*. The numerical data were analyzed by Student's t-test using GraphPad Prism 8. Values were presented as mean ± SD, ** $p < 0.01$.

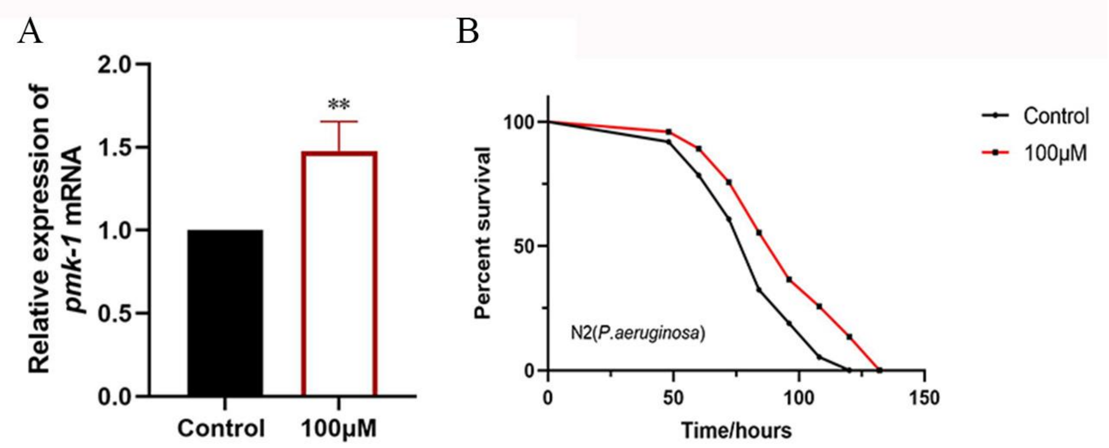

Table S4. Effects of EA on the lifespan of N2 C.elegans cultured with *Pseudomonas aeruginosa*. *p* value compared to the control (0 μM) group.

Genotype	Treatment	Mean (Hours)	±SD	Percent Change （%）	*p* value (log-rank significance)
pmk-1	0 μM	82.54	19.46		
	100 μM	95.03	23.57	15.13	< 0.001
